# Adverse sexual and reproductive health outcomes in female adolescents with ADHD and their associations with ADHD medication: a Swedish register-based study

**DOI:** 10.1136/bmjment-2026-302883

**Published:** 2026-10-12

**Authors:** Helena Kopp Kallner, Xinchen Wang, Le Zhang, Taalke Maria Sitter, Lisa B Thorell, Charlotte Skoglund, Zheng Chang

**Affiliations:** 1 Department of Clinical Sciences, Karolinska Institutet, Stockholm, Sweden; 2 Department of Obstetrics and Gynaecology, Danderyd Hospital, Stockholm, Sweden; 3 Department of Clinical Neuroscience, Karolinska Institutet, Stockholm, Sweden; 4 Department of Medical Epidemiology and Biostatistics, Karolinska Institute, Stockholm, Sweden; 5 School of Public Health, Shenzhen University, Shenzhen, Guangdong, China; 6 Department of Women's and Children's Health, Uppsala University, Uppsala, Sweden

## Abstract

**Background:**

Females with attention-deficit/hyperactivity disorder (ADHD) are often diagnosed later than males and may experience disproportionate sexual and reproductive health risks. Evidence on whether ADHD medication mitigates adverse sexual and reproductive health (SRH) outcomes in female adolescents with ADHD remains limited.

**Objective:**

To examine the association between ADHD diagnosis, medication treatment and adverse SRH outcomes in adolescent girls.

**Methods:**

We conducted a population-based cohort study in Stockholm, Sweden, including females aged 4–20 years with an incident ADHD diagnosis or medication between 2011 and 2021. Individuals with ADHD were matched 1:5 to peers without ADHD based on sex, birth year and socioeconomic status. Adverse SRH outcomes were defined as sexually transmitted infections (STIs) or unwanted pregnancy. Cox proportional hazards models estimated associations between ADHD and adverse SRH outcomes. Within the ADHD cohort, we conducted a nested case-control study to assess associations between ADHD medication use and adverse SRH outcomes using conditional logistic regression.

**Results:**

Among 18 776 female adolescents with ADHD and 93 880 matched controls, 1787 (29.3 per 1000 person-years) and 4779 (15.2 per 1000 person-years) had an adverse SRH outcome recorded, respectively. ADHD was associated with an increased risk of adverse SRH outcomes (adjusted HR 1.70, 95% CI 1.59 to 1.81), with stronger associations for unwanted pregnancy than for STIs. Risk remained elevated in individuals without psychiatric comorbidity and was highest for those with comorbid substance use disorder. Longer ADHD medication use was associated with a progressively lower risk of adverse SRH outcomes (adjusted OR 0.64, 95% CI 0.44 to 0.94 for >5 years versus ≤1 year of treatment).

**Conclusion:**

Female adolescents with ADHD have an increased risk of adverse SRH outcomes, independent of comorbidity. Longer ADHD medication duration is associated with lower risks.

**Clinical implications:**

The findings emphasise the importance of timely diagnosis and sustained management of ADHD in girls to reduce adverse SRH outcomes.

**WHAT IS ALREADY KNOWN ON THIS TOPIC**

- Previous studies and systematic reviews have shown that individuals with attention-deficit/hyperactivity disorder (ADHD) are at increased risk of adverse sexual and reproductive health outcomes, including sexually transmitted infections and early or unwanted pregnancy.
- Most existing evidence is derived from studies limited by small sample sizes, voluntary participation, reliance on self-reported ADHD symptoms and sexual behaviour, and inadequate control for potential confounders.

**WHAT THIS STUDY ADDS**

- ADHD was associated with a nearly twofold increased risk of adverse sexual and reproductive health outcomes after adjusting for potential confounders. The association persisted in the absence of comorbid anxiety, depression or substance use disorders.
- Longer duration of ADHD medication use was associated with progressively lower risks of these outcomes.

**HOW THIS STUDY MIGHT AFFECT RESEARCH, PRACTICE OR POLICY**

- Psychiatrists and paediatricians could consider incorporating counselling and screening for sexual health into the management of adolescents with ADHD.
- The association between ADHD medication use and decreased risks suggests the clinical importance of treatment continuity and adherence.

## Background

 Studies show that girls and women with attention-deficit/hyperactivity disorder (ADHD) are detected, diagnosed and treated later than males, and that barriers to ADHD diagnosis and treatment include the intersection between being female and low socioeconomic status.^1–4^ Delayed diagnosis may add to the disproportionate female-specific burden of adverse reproductive health outcomes.^5^

Adverse sexual and reproductive health (SRH) outcomes due to impulsivity and poor decision-making are a well-established problem among youth with ADHD. The consequences include increased risk of becoming victimised, sexually assaulted, contracting sexually transmitted infections (STIs) and, for women specifically, using emergency contraception, using less regular contraception and experiencing unwanted pregnancies.^6–9^ There are several female-specific consequences of SRH outcomes, such as severe pelvic inflammatory disease, chronic pain, future infertility, ectopic pregnancies, induced abortion or giving birth.^10 11^

Randomised controlled clinical trials and observational register-based studies show that pharmacological treatment with stimulant and non-stimulant medications effectively reduces ADHD symptom levels and negative physical outcomes in ADHD, such as fractures, unspecified injuries and emergency visits in children or adolescents.^12^ Two previous large studies suggest that pharmacological treatment for ADHD reduces the risk for adverse SRH outcomes in women with ADHD,^13 14^ while one smaller study (n≈2000) found no association.^15^

To improve female sexual health in a growing and previously underserved group of vulnerable young women, we need to further assess the association between ADHD medication and adverse outcomes in females across the lifespan.^5^ Generalisability of previous studies on SRH outcomes in women may be limited by differences in cultural views and stigma related to female and adolescent sexuality, contraception and induced abortion. Therefore, we performed a register-based prospective cohort study to explore the impact of ADHD and comorbidities on adverse SRH outcomes (ie, STI, unwanted pregnancy) and the role of ADHD medication treatment in a population of Swedish female adolescents with ADHD.

## Methods

### Data sources

The data used in this study were obtained from the Regional Healthcare Data Warehouse of Region Stockholm (VAL), which contains comprehensive information on all individual contacts with the publicly funded healthcare system in Stockholm County.^1 16^ In Sweden, healthcare is almost exclusively publicly funded, and each resident is assigned a unique personal identification number, enabling accurate linkage across registers.^17^ The VAL contains all consultations in primary and secondary care (ie, outpatient care) and all hospitalisations (ie, inpatient care). Data for primary care are available from 2003, whereas data for secondary care and hospitalisations date back to 1987, based on the International Classification of Diseases (ICD) in its ninth (ICD-9; 1987–1996) and 10th (ICD-10; since 1997) revisions.^1 16^ Information on prescribed medications was obtained from the Swedish Prescribed Drug Register, which contains all medications dispensed in Sweden and is coded by the Anatomical Therapeutic Chemical (ATC) classification.^18^ Data on medications dispensed in ambulatory care settings have been included since July 2010.^1^ The VAL database captures ADHD diagnoses made within publicly funded psychiatric care. However, individuals diagnosed in private care are still captured in the Prescribed Drug Register if they are prescribed ADHD medications, since all such medications are covered by the public reimbursement system. VAL also provides demographic data, including age, sex, migration status and death. Biological sex at birth is used to identify female individuals.

### Study design and participants

We identified all females residing in the Stockholm Region aged 4–20 years who received an incident ADHD diagnosis or ADHD medication dispensation between 1 January 2011 and 31 December 2021. ADHD was defined as a recorded diagnosis of ADHD (ICD-10: F90) and/or filling a prescription for any stimulant or non-stimulant ADHD medication (Amphetamine (ATC: N06BA01), Dexamphetamine (N06BA02), Methylphenidate (N06BA04), Atomoxetine (N06BA09), Lisdexamphetamine (N06BA12) and Guanfacine (C02AC02)). Incident ADHD medication dispensation was defined as a dispensation after at least 18 months without any ADHD medication dispensation.^19^ This approach to identify individuals with ADHD has been commonly used in Swedish register-based studies.^19–21^ Individuals who had resided in Stockholm for less than 1 year, emigrated, died or had a history of adverse SRH outcomes were excluded from the study (online supplemental eFigure 1). Each individual with ADHD was matched with up to five individuals without ADHD from the general population based on sex, birth year and socioeconomic status. Baseline for the matched cohort was defined as the date of incident ADHD diagnosis or medication dispensation, whichever came first. The matched cohort was followed from baseline until the date of adverse SRH outcomes (including STI, induced abortion and unwanted pregnancy; see definition below), death, first emigration, reaching 20 years or 31 December 2021, whichever came first.

We used a nested case-control design to examine the association between ADHD medication use and adverse SRH outcomes within the cohort of individuals with ADHD. Cases were defined as an incident of any adverse SRH outcome during follow-up. For each case, the date of their SRH event was assigned as the index date. Using incidence density sampling,^22^ up to five controls without SRH outcomes were randomly selected for each case from the base cohort of individuals with ADHD (online supplemental eFigure 1). Controls were eligible if they were alive, residing in Stockholm, and free of the studied SRH outcomes at the time their matched case experienced the SRH event, with the index date set as the date of the SRH event of the matched case. The matching variables included birth year, year of first ADHD diagnosis (or first dispensed ADHD medication) and socioeconomic status, ensuring that cases and controls had the same follow-up duration from baseline to index date.

### Measures

Cumulative ADHD medication use among girls with ADHD was defined as the total duration of time (in years) during which individuals were covered by ADHD medication between baseline and the index date. In line with previous Swedish psychiatric medication research, two dispensations occurring within 120 days of each other were considered part of the same continuous treatment period.^23^ The total cumulative duration of ADHD medication use was then obtained by aggregating these treatment periods across the follow-up period.

SRH outcomes were defined as the occurrence of a recorded STI or a visit for unwanted pregnancy before the age of 20 years (ICD/ATC codes in online supplemental eTable 1). The definition of STI included diagnoses of viral hepatitis, HIV, chlamydia, gonorrhoea, syphilis, condyloma and other forms of STIs, as well as dispensation of doxycycline (excluding those with a diagnosis of acne (L700–L709), other spirochaetal infections (A69, including Lyme borreliosis), rosacea (L71) or respiratory tract infection (J00–J06 and J12–J18) recorded within 30 days before the dispensation). Unwanted pregnancy included diagnosis of problems related to unwanted pregnancy and induced abortion.

### Statistical analysis

To examine the association between ADHD and risk of adverse SRH outcomes, we used stratified Cox proportional hazard regression models to estimate HRs with 95% CIs, with attained age as the underlying time scale. Crude HRs were adjusted for all matching variables by design. Adjusted HRs were additionally controlled for comorbidities (obesity, sleep problems, anxiety disorder, autism spectrum disorder, conduct disorder, depressive disorder, eating disorders, intellectual disability, personality disorders, severe mental illness and substance use disorder) and concomitant medications (opioids, antiepileptic drugs, antipsychotics, anxiolytics, hypnotics or sedatives and antidepressants) any time before baseline, as well as healthcare utilisation (any hospitalisations for psychiatry, any hospitalisations for non-psychiatric reason, any outpatient visit for psychiatry and any outpatient visit for non-psychiatric reason) 1 year before baseline (see ICD/ATC codes in online supplemental eTable 1). In addition, we examined the associations in girls with ADHD and comorbid anxiety, depression or substance use disorder with girls without ADHD and without the corresponding comorbidity as the reference group, as these comorbidities are common in this population and may further modify the risk of SRH outcomes.^24^ This analysis differs from the primary model, which adjusts for comorbidities and uses all girls without ADHD, regardless of comorbidity status, as the reference group.

To examine the associations between cumulative duration of ADHD medication use and risk of adverse SRH outcomes, we conducted conditional logistic regressions to estimate ORs. The ORs were adjusted for the same set of covariates described above. The duration of ADHD medication use was first modelled as categorical (ie, no-use, 0 to ≤1 (reference), 1 to ≤2, 2 to ≤3, 3 to ≤5 and >5 years use). We further applied restricted cubic splines to flexibly model potential nonlinear associations between ADHD medication use and SRH outcomes across the full exposure range up to 10 years.^25^ To reduce potential unmeasured confounding between medication users and nonusers, this analysis was restricted to individuals with at least one dispensation of ADHD medication. We evaluated models with three, four and five knots and selected the three-knot specification based on the lowest Bayesian Information Criterion, indicating the best model fit.

Our study was reported in accordance with the Strengthening the Reporting of Observational Studies in Epidemiology (STROBE) statement.^26^

## Results

We identified 22 611 girls aged 4–20 years with a diagnosis of ADHD or dispensed ADHD medication. After applying exclusion criteria, 18 776 girls remained eligible for matching (online supplemental eFigure 1). These girls constituted the ADHD cohort and were matched 1:5 with 93 880 girls without ADHD from the general population. Among the 18 776 girls with ADHD, 1787 individuals had an incident SRH outcome of interest by the age of 20 years, and they were matched to 8909 controls without SRH outcomes.

Table 1 presents baseline characteristics of the matched cohort of girls with and without ADHD. The median age at baseline was 13 years (IQR 10–16). Compared with girls without ADHD, girls with ADHD had a higher prevalence of obesity and psychiatric comorbidity before baseline. Girls with ADHD were also more likely to have been prescribed opioids and psychotropic medications before baseline and to have higher rates of psychiatric and non-psychiatric hospitalisations and outpatient psychiatric visits. Among girls with ADHD included in the nested case-control analysis, a higher proportion of cases with SRH outcomes had sleep problems, conduct disorder and substance use disorder, as well as prior use of opioids, anxiolytics, hypnotics or sedatives than the controls (table 1). Cases with SRH outcomes also had higher rates of psychiatric and non-psychiatric hospitalisations, as well as psychiatric outpatient visits in the year before ADHD diagnosis.

**Table 1:** Characteristics of girls with ADHD and their matched controls during 2011–2021

Characteristics	ADHD (n=18 776) No. (%)	Without ADHD (n=93 880) No. (%)	ADHD with adverse SRH outcomes (cases) (n=1787) No. (%)	ADHD without adverse SRH outcomes (controls) (n=8909) No. (%)
Age (years) at first ADHD diagnosis/medication (median (IQR))*	13 (10–16)	13 (10–16)	15 (14–17)	15 (14–17)
Age at SRH outcomes outcome diagnosis†	19 (17–20)	19 (17–21)	18 (17–19)	18 (17–19)
Socioeconomic status				
High	5061 (27.0)	25 305 (27.0)	436 (24.4)	2175 (24.4)
Middle	7911 (42.1)	39 555 (42.1)	758 (42.4)	3779 (42.4)
Low	5789 (30.8)	28 945 (30.8)	593 (33.2)	2955 (33.2)
Missing	15 (0.1)	75 (0.1)	0	0
Diagnosis before baseline				
Obesity	1171 (6.2)	2606 (2.8)	78 (4.4)	637 (7.2)
Sleep problems	2132 (11.4)	3011 (3.2)	176 (9.8)	819 (9.2)
Anxiety disorder	3794 (20.2)	5580 (5.9)	376 (21.0)	1995 (22.4)
Autism spectrum disorder	1146 (6.1)	1778 (1.9)	63 (3.5)	545 (6.1)
Conduct disorder	643 (3.4)	504 (0.5)	93 (5.2)	279 (3.1)
Depressive disorder	2277 (12.1)	2694 (2.9)	280 (15.7)	1394 (15.6)
Eating disorders	540 (2.9)	1203 (1.3)	55 (3.1)	352 (4.0)
Intellectual disability	223 (1.2)	704 (0.7)	6 (0.3)	118 (1.3)
Severe mental illness‡	77 (0.4)	118 (0.1)	9 (0.5)	48 (0.5)
Substance use disorder	676 (3.6)	834 (0.9)	173 (9.7)	430 (4.8)
Medication before baseline				
Opioids	779 (4.1)	2149 (2.3)	124 (6.9)	447 (5.0)
Antiepileptic drugs	359 (1.9)	759 (0.8)	28 (1.6)	179 (2.0)
Antipsychotics	460 (2.4)	521 (0.6)	39 (2.2)	256 (2.9)
Anxiolytics, hypnotics or sedatives	4622 (24.6)	5810 (6.2)	442 (24.7)	2105 (23.6)
Antidepressants	2576 (13.7)	2799 (3.0)	295 (16.5)	1637 (18.4)
Healthcare utilisation 1 year before baseline				
Any hospitalisations for psychiatry	426 (2.3)	453 (0.5)	83 (4.6)	246 (2.8)
Any hospitalisations for non-psychiatric reason	410 (2.2)	883 (0.9)	66 (3.7)	215 (2.4)
Any outpatient visits for psychiatry	3525 (18.8)	4168 (4.4)	161 (9.0)	760 (8.5)
Any outpatient visits for non-psychiatric reason	306 (1.6)	2090 (2.2)	7 (0.4)	31 (0.3)

* Age at first ADHD diagnosis, or for controls in the matched cohort, age at the time of matching to the ADHD case.

† Age at first adverse sexual and reproductive health outcomes, or for controls in the case-control study, age at the time of matching to the SRH outcomes case.

‡ Severe mental illness: bipolar disorder and/or schizophrenia.

ADHD, attention deficit hyperactivity disorder; SRH, sexual and reproductive health.

During follow-up, 1787 girls with ADHD (incidence rate (IR), 29.3 per 1000 person-years) and 4779 girls without ADHD (IR 15.2 per 1000 person-years) had an SRH outcome of interest recorded. The frequency of individual SRH events in the matched cohort is presented in online supplemental eTable 2. Girls with ADHD had a twofold increased crude risk of SRH outcomes compared with matched girls without (HR 2.00, 95% CI 1.89 to 2.12; table 2). This association remained significant after adjusting for somatic and psychiatric comorbidities, prior medication use and healthcare utilisation (adjusted HR (aHR) 1.70, 95% CI 1.59 to 1.81). When examining specific SRH outcomes, the adjusted HRs were 1.60 (95% CI 1.48 to 1.73) for STI and 1.99 (95% CI 1.83 to 2.16) for unwanted pregnancy, indicating a stronger association for unwanted pregnancy than for STI (table 2).

**Table 2:** Risk of adverse SRH outcomes among girls with ADHD compared with those without ADHD

	With ADHD (n=18 776)		Without ADHD (n=93 880)		HR*	Adjusted HR†
	Cases, (IR)‡	Person years	Cases, (IR)‡	Person years		
Any SRH outcomes	1787 (29.3)	61 086	4779 (15.2)	314 499	2.00 (1.89–2.12)	1.70 (1.59–1.81)
STI	1080 (17.3)	62 464	2973 (9.4)	317 421	1.88 (1.75–2.02)	1.60 (1.48–1.73)
STI (only diagnosis)	377 (6.0)	63 611	1018 (3.2)	320 654	1.89 (1.68–2.13)	1.57 (1.37–1.80)
Unwanted pregnancy	1071 (17.2)	62 385	2412 (7.6)	318 427	2.37 (2.20–2.55)	1.99 (1.83–2.16)

* Matched on birth year and socioeconomic status.

† HRs were adjusted for obesity, sleep problems, anxiety disorder, autism spectrum disorder, conduct disorder, depressive disorder, eating disorders, intellectual disability, personality disorders, severe mental illness, substance use disorder, opioids, antiepileptic drugs, antipsychotics, anxiolytics, hypnotics or sedatives, antidepressants, any hospitalisations for psychiatry, any hospitalisations for non-psychiatric reason, any outpatient visit for psychiatry and any outpatient visit for non-psychiatric reason.

‡ Incidence rates per 1000 person years.

ADHD, attention deficit hyperactivity disorder; IR, incidence rate; SRH, sexual and reproductive health; STI, sexually transmitted infection.

Further analyses stratified by comorbidities of anxiety, depression and substance use disorder (table 3) showed that girls with ADHD, even without any of these comorbidities, still had an increased risk of adverse SRH outcomes compared with girls without ADHD (aHR 2.00, 95% CI 1.86 to 2.15). A similar magnitude of risk was observed for those with comorbid depression (aHR 2.05, 95% CI 1.58 to 2.65), whereas the elevated risk appeared to be lower in those with comorbid anxiety (aHR 1.61, 95% CI 1.34 to 1.93) and, in contrast, markedly higher among girls with ADHD and comorbid substance use disorder (aHR 4.68, 95% CI 3.38 to 6.48) compared with those without ADHD.

**Table 3:** Association between ADHD and adverse SRH outcomes in girls with comorbid anxiety, depression or substance use disorder

	Cases (IR)*	Person years	HR†	aHR‡
ADHD only	1182 (24.0)	49 221	2.09 (1.95–2.24)	2.00 (1.86–2.15)
ADHD and anxiety	203 (36.1)	5620	1.89 (1.60–2.23)	1.61 (1.34–1.93)
ADHD and depression	109 (52.2)	2090	2.38 (1.88–3.02)	2.05 (1.58–2.65)
ADHD and SUD	96 (122.6)	783	5.87 (4.30–8.02)	4.68 (3.38–6.48)
ADHD with more than one comorbidity§	197 (58.4)	3373	2.50 (2.09–2.98)	2.04 (1.62–2.55)
No ADHD or comorbidity§	4015 (13.5)	297 118	1 (Reference)	1 (Reference)

* Incidence rates per 1000 person years.

† Matched on birth year and socioeconomic status.

‡ HRs were adjusted for covariates other than anxiety disorder, depressive disorder and substance use disorder: obesity, sleep problems, autism spectrum disorder, conduct disorder, eating disorders, intellectual disability, personality disorders, severe mental illness, opioids, antiepileptic drugs, antipsychotics, anxiolytics, hypnotics or sedatives, antidepressants, any hospitalisations for psychiatry, any hospitalisations for non-psychiatric reason, any outpatient visit for psychiatry and any outpatient visit for non-psychiatric reason.

§ The comorbidity refers to anxiety disorder, depressive disorder and substance use disorder.

ADHD, attention deficit hyperactivity disorder; SRH, sexual and reproductive health; STI, sexually transmitted infection; SUD, substance use disorder.

Longer cumulative duration of ADHD medication use was associated with a lower risk of adverse SRH outcomes (table 4). With ≤1 year of medication use as the reference category, longer ADHD medication use was associated with lower odds of SRH outcomes, with a clear dose-response pattern. The lowest risk was observed among girls with more than 5 years of cumulative ADHD medication use (adjusted OR (aOR) 0.64, 95% CI 0.44 to 0.94). Girls with no ADHD medication use also had a lower risk of the studied SRH outcomes than those with ≤1 year of medication use, likely reflecting unmeasured differences compared with medication users. The restricted cubic spline model in ADHD medication users supported a dose-response association, showing progressively lower risk of adverse SRH outcomes with longer duration of medication use (online supplemental eFigure 2).

**Table 4:** Association between cumulative duration of ADHD medication use and risk of SRH outcomes in girls with ADHD

Cumulative years of ADHD medication use	Cases, No. (%) (n=1787)	Controls, No. (%) (n=8909)	OR (95% CI)*	aOR (95% CI)†
No use	263 (14.7)	1701 (19.1)	0.65 (0.56 to 0.76)	0.67 (0.57 to 0.79)
0 to ≤1	858 (48.0)	3746 (42.1)	1 (Reference)	1 (Reference)
1 to ≤2	371 (20.8)	1737 (19.5)	0.93 (0.81 to 1.07)	0.94 (0.81 to 1.08)
2 to ≤3	145 (8.1)	804 (9.0)	0.76 (0.62 to 0.93)	0.77 (0.63 to 0.95)
3 to ≤5	108 (6.0)	668 (7.5)	0.65 (0.52 to 0.83)	0.65 (0.51 to 0.83)
>5	42 (2.4)	253 (2.8)	0.64 (0.44 to 0.93)	0.64 0.44 to 0.94)

* Based on cases and controls matched on birth year, socioeconomic status and year of ADHD diagnosis.

† ORs were adjusted for obesity, sleep problems, anxiety disorder, autism spectrum disorder, conduct disorder, depressive disorder, eating disorders, intellectual disability, personality disorders, severe mental illness, substance use disorder, opioids, antiepileptic drugs, antipsychotics, anxiolytics, hypnotics or sedatives, antidepressants, any hospitalisations for psychiatry, any hospitalisations for non-psychiatric reason, any outpatient visit for psychiatry and any outpatient visit for non-psychiatric reason.

ADHD, attention deficit hyperactivity disorder; SRH, sexual and reproductive health.

## Discussion

In this population-based study, we found that female adolescents with ADHD had an almost doubled risk of adverse SRH outcomes compared with those without ADHD. The association remained significant in the fully adjusted model and was independent of psychiatric comorbidity, while comorbid substance use disorder was associated with the highest risk. We found a stronger association for unwanted pregnancy than for STIs. In addition, longer duration of ADHD medication use was associated with progressively lower odds of SRH outcomes.

Our study expands previous research using national registers that have shown a decreased risk for adverse outcomes during periods on active medication in several areas, including accidents, depression and suicide^27–31^; as well as a 40% reduction in the risk of criminal behaviour for women specifically.^32^ However, when specifically studying SRH outcomes, a previous study by Chen *et al* using the National Health Insurance Research Database in Taiwan to compare adolescent and young adult males and females with ADHD with age-matched and sex-matched controls, presented results that differ from ours.^13^ Chen *et al* found that both short-term (>30 days to <364 days) and long-term use (> 365 days) of ADHD medication was associated with a reduction in STI in men but not in women. Overall, SRH events were less frequent in the Taiwanese than in our Swedish data across all outcomes. Differences in setting and culture may explain these differences. Taiwanese females have fewer lifetime sex partners than males, and both sexes have significantly fewer lifetime sex partners than same-aged individuals in the Nordic countries.^33 34^ There may also be more asymptomatic infections diagnosed in Sweden, as youth clinics are easily accessible and provide free, confidential (ie, guardians do not have to be notified) testing for both STIs and pregnancy. Indeed, Swedish youth centres encourage STI testing after every unprotected intercourse, and tests can easily be ordered online. Furthermore, abortion is free and available without guardians having to be notified. Thus, our results confirm that ADHD medication also reduces diagnoses of SRH outcomes in a liberal setting with low stigma.

The increased risk for the studied SRH outcomes in individuals with ADHD is associated both with an increased perception of benefits (such as enjoyment) as well as a reduced perception of risk (such as STIs and unwanted pregnancy).^35^ Stimulant medication may have a positive effect on executive function - an effect that may be even more pronounced in a cultural setting with more liberal views on female sexuality. In addition, we could see a further elevated risk of SRH outcomes with substance use disorder. Duration of stimulant medication use has previously been shown to be associated with a lower risk of substance abuse.^36^ Thus, reduced risky alcohol and drug use may also contribute to the observed association of long duration of ADHD medication use and reduced risks of SRH outcomes.

We included visits for unwanted pregnancy and abortion in our study, with abortion serving as a proxy for unwanted pregnancy. In a setting with a liberal view on abortion, we considered unwanted pregnancy, instead of abortion, as the underlying adverse reproductive outcome. Unwanted pregnancy is closely linked to contraceptive use. Due to poor executive functioning, women with ADHD may fail to use coitus-timed contraception (eg, male condoms) consistently, and they have been shown to have lower use of any form of contraception.^8^ Women with ADHD also more often experience depression with use of short-acting contraception (pills, patch and ring),^37^ which may lead to discontinuation and subsequent increased risk of unplanned and unwanted pregnancy. Adequate symptom control with ADHD medication could possibly increase consistent use of coitus-timed contraception, and therefore not only reduce SRH outcomes but also the consequences of SRH outcomes.

Comorbid substance use disorder in individuals with ADHD has been shown to increase risk for several adverse outcomes.^38^ This association holds for adverse SRH outcomes as well. A study of ADHD symptom levels (ie, not a formal ADHD diagnosis) in French students found no association between occasional substance use and SRH outcomes,^8^ suggesting that it might be the substance use disorder or substance abuse, rather than the effect of the substance per se, that is associated with the increased risk for SRH outcomes. Regarding comorbid anxiety, we observed a lower HR for SRH outcomes among girls with ADHD and anxiety than among those with ADHD alone. In contrast, our data showed a trend towards reduced risk of SRH outcomes with comorbid anxiety. Given the wide CI, this finding should be considered hypothesis-generating. Further studies are needed to determine whether this pattern can be replicated for SRH outcomes.

The regional healthcare register used in this study is highly granular and contains data from both primary and secondary care. Medication exposure is reported from filled prescriptions, and as in all register-based studies, we cannot ascertain whether they were taken as prescribed. However, the longitudinal design enabled the establishment of the temporal correlation between ADHD medication use and risk for SRH outcomes. The high-quality register data also enabled matching by SES and adjustment for a wide range of comorbidities that could affect SRH outcomes. Identification of ADHD diagnosis was based on an ICD-10 record of F90 or dispensed prescription of ADHD medication. We could not control for ADHD symptom severity, which may partly explain the lower risk of SRH outcomes in those with no ADHD medication use as compared with those treated for ≤1 year. More generally, girls who accumulated longer durations of medication treatment may differ from those who accumulated little or no treatment on unmeasured determinants of treatment persistence, including ADHD severity, family support and health-seeking behaviour. These differences may have contributed to the observed association between longer medication use and lower risks of SRH outcomes. We studied first-time adverse SRH events, excluding individuals with prior outcomes. Thus, we could not assess associations with recurrent SRH outcomes. Some misclassifications of doxycycline-defined STI may remain because treatment indications are not always recorded. Yet, the sensitivity analysis restricted to STI diagnoses only yielded similar results. In addition, as in all register-based studies, the study captured only diagnoses and outcomes recorded through healthcare contact. Girls with ADHD had more frequent healthcare contact than controls at baseline. It cannot be excluded that this could lead to more complete ascertainment of SRH outcomes in the ADHD group.

In conclusion, women with ADHD are at increased risk of adverse SRH outcomes. The association is more prominent for unwanted pregnancy than STIs and independent of psychiatric comorbidity. Lower risk of SRH outcomes was associated with ADHD medication use in female adolescents, with greater reductions associated with longer medication use. Timely diagnosis and adequate treatment of females with ADHD can reduce negative sexual and reproductive health outcomes both in a short- and long-term perspective.

## Supplementary material

10.1136/bmjment-2026-302883 online supplemental file 1

## Data Availability

Data may be obtained from a third party and are not publicly available.
