# Supplementary material for: Adverse sexual and reproductive health outcomes in female adolescents with ADHD and their associations with ADHD medication: a Swedish register-based study
Source: BMJ Ment Health. 2026 Oct 12;29(1):e302883. doi: 10.1136/bmjment-2026-302883 (PMC13662076; doi:10.1136/bmjment-2026-302883)
Supplement: Supplementary file 1 [file bmjment-29-1-s001.pdf]

Supplementary Materials: Adverse sexual and reproductive health outcomes in female adolescents with ADHD and their associations with ADHD medication: A Swedish register-based study

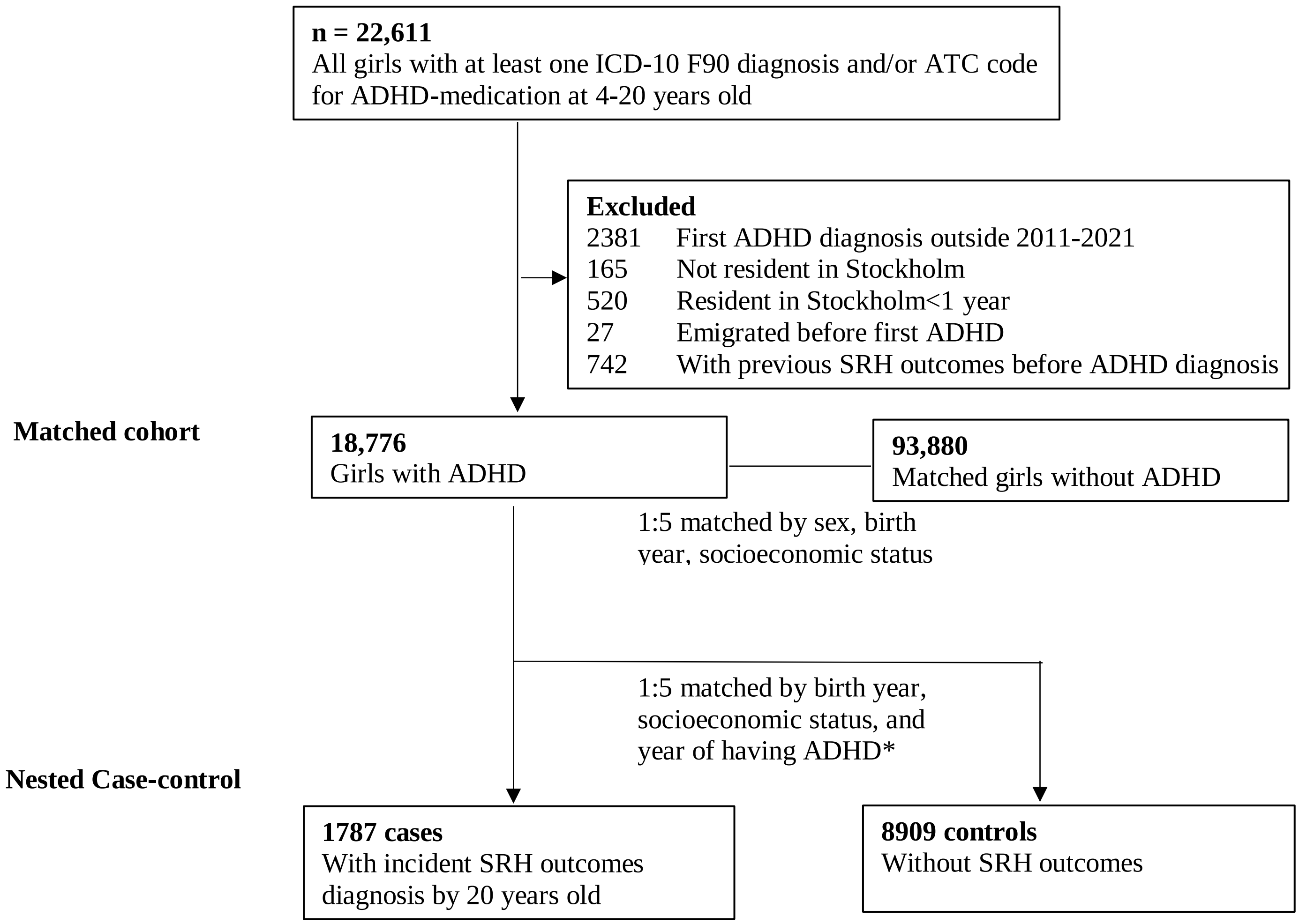

eFigure 1. Inclusion of study population.

* Of the 1787 individuals with adverse sexual and reproductive health outcomes (SRH outcomes), 1776 were 1:5 matched with 8880 controls. The remaining 11 cases were matched with fewer than five controls, contributing 29 additional controls. The total number of controls across all matched cases was therefore 8909.

eTable 1.ICD/ATC Codes for adverse SRH outcomes and comorbidities.

**Condition**	**ICD-10/ATC Codes**
**Any STI**	Diagnosis: A5, A60, A63, A64, B15-19, B20-24<br>Medication: J01AA02
Viral hepatitis	B15, B16, B17, B18, B19
HIV	B20, B21, B22, B23, B24
Chlamydia and	A56
Gonorrhoea	A54
Syphilis	A51, A52, A53
Condyloma	A63
Doxycycline	J01AA02
**Unwanted pregnancy**	O04, Z64
Obesity	E65, E66
Sleep problems	F51, G47.0- G47.4, G47.8, G47.9
Anxiety disorder	F40-F41
Autism spectrum disorder	F84
Bipolar disorder	F30-F31
Conduct disorder	F91
Depressive disorder	F32-33
Eating disorders	F50
Intellectual disability	F7
Schizophrenia	F2
Substance use disorder	F10-F19

eTable 2. Frequency of SRH outcomes events in the matched cohort.

Condition	With ADHD (n=18,776), No. (%)	Without ADHD (n=93,880), No. (%)
**Any STI**	1080 (5.7)	2973 (3.2)
Doxycycline	703 (3.7)	1,955 (2.1)
Viral hepatitis	6 (0.0)	26 (0.0)
HIV	<5 (0.0)	<5 (0.0)
Chlamydia	121 (0.6)	297 (0.3)
Gonorrhea	56 (0.3)	133 (0.1)
Syphilis	<5 (0.0)	<5 (0.0)
Herpes	138 (0.7)	400 (0.4)
Condyloma	53 (0.3)	151 (0.2)
Other STI	<5 (0.0)	7 (0.0)
**Unwanted pregnancy**	1071 (5.7)	2412 (2.6)

Abbreviation: SRH outcomes, adverse sexual and reproductive health outcomes; HIV, human immunodeficiency viruses infection; STI, sexually transmitted infection

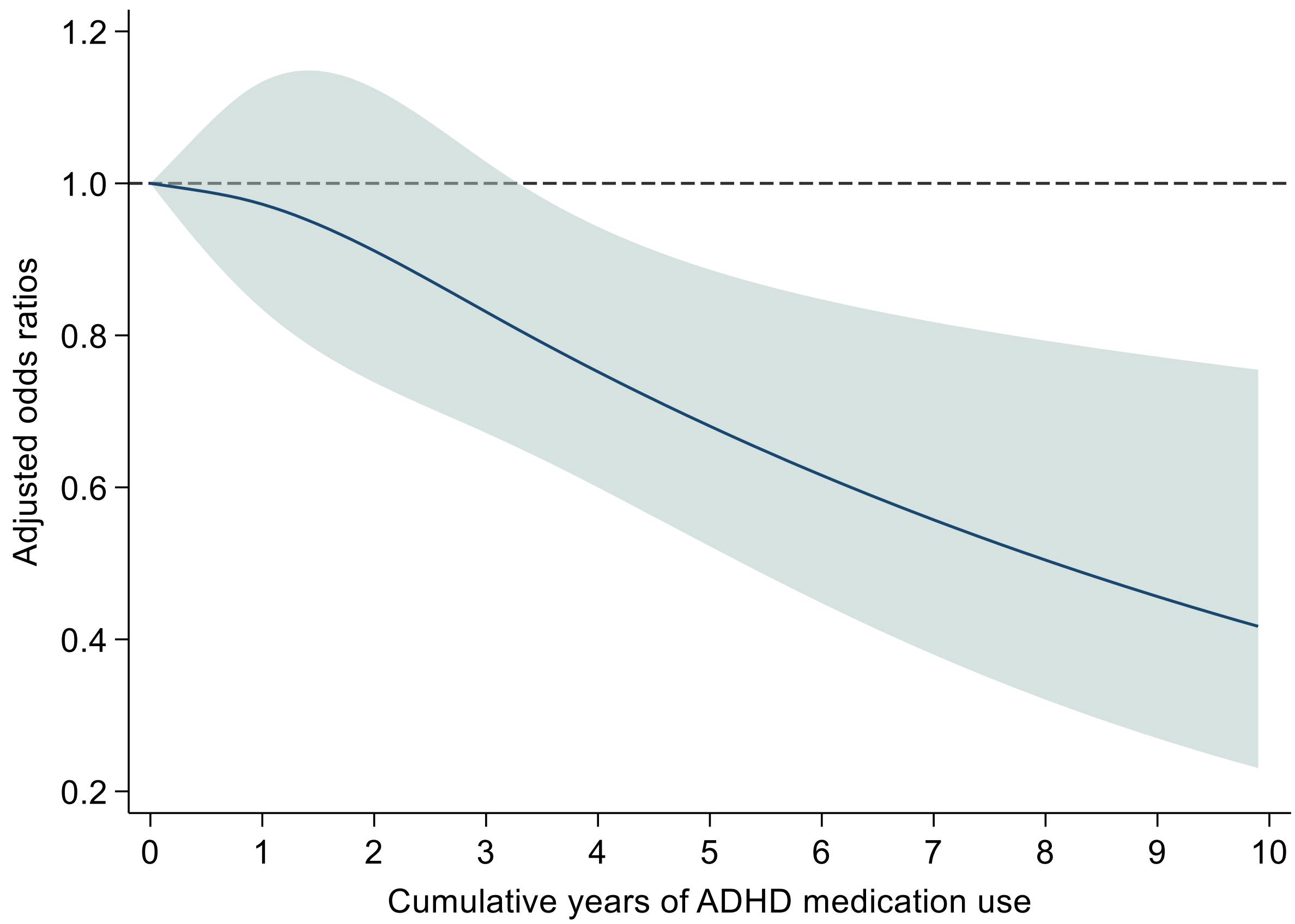

eFigure 2. Association between cumulative ADHD medication use and risk of SRH outcomes among to ADHD medication users

The solid lines represent the adjusted odds ratios, and the shaded areas represent the 95% CIs. In restricted cubic splines analysis, knots were placed at the 10th, 50th, and 90th percentiles of ADHD medication use.
